# The frequency of Tay-Sachs disease causing mutations in the Brazilian Jewish population justifies a carrier screening program

**DOI:** 10.1590/S1516-31802001000400007

**Published:** 2001-07-07

**Authors:** Roberto Rozenberg, Lygia da Veiga Pereira

**Keywords:** Tay-Sachsdisease, Genetic screening, HEXA gene, Jewish population, Molecular diagnosis, Doença de Tay-Sachs, Triagem genética, Gene HEXA, População judaica, Diagnóstico molecular

## Abstract

**CONTEXT:**

Tay-Sachs disease is an autosomal recessive disease characterized by progressive neurologic degeneration, fatal in early childhood. In the Ashkenazi Jewish population the disease incidence is about 1 in every 3,500 newborns and the carrier frequency is 1 in every 29 individuals. Carrier screening programs for Tay-Sachs disease have reduced disease incidence by 90% in high-risk populations in several countries. The Brazilian Jewish population is estimated at 90,000 individuals. Currently, there is no screening program for Tay-Sachs disease in this population.

**OBJECTIVE:**

To evaluate the importance of a Tay-Sachs disease carrier screening program in the Brazilian Jewish population by determining the frequency of heterozygotes and the acceptance of the program by the community.

**SETTING:**

Laboratoryof Molecular Genetics – Institute of Biosciences – Universidade de São Paulo.

**PARTICIPANTS:**

581 senior students from selected Jewish high schools.

**PROCEDURE:**

Molecularanalysis of Tay-Sachs disease causing mutations by PCR amplification of genomic DNA, followed by restriction enzyme digestion.

**RESULTS:**

Among 581 students that attended educational classes, 404 (70%) elected to be tested for Tay-Sachs disease mutations. Of these, approximately 65% were of Ashkenazi Jewish origin. Eight carriers were detected corresponding to a carrier frequency of 1 in every 33 individuals in the Ashkenazi Jewish fraction of the sample.

**CONCLUSION:**

The frequency of Tay-Sachs disease carriers among the Ashkenazi Jewish population of Brazil is similar to that of other countries where carrier screening programs have led to a significant decrease in disease incidence. Therefore, it is justifiable to implement a Tay-Sachs disease carrier screening program for the Brazilian Jewish population.

## RESUMO

**CONTEXTO:**

A doença de Tay-Sachs é uma doença autossômica recessiva caracterizada por uma degeneração neurológica progressiva, fatal na primeira infância. Na população judaica Ashkenazita a incidência da doença é de um para cada 3.500 nascimentos, e a freqüência de portadores é de um para cada 29 indivíduos. Programas de triagem de portadores da doença de Tay-Sachs reduziram a incidência da doença em 90% nas populações em risco de diversos países. A população judaica brasileira é estimada em 90.000 indivíduos. Atualmente não há programa de triagem populacional da doença de Tay-Sachs nessa população.

**OBJETIVO:**

Avaliar a importância de um programa de triagem de portadores da doença de Tay-Sachs na população judaica brasileira, através do estabelecimento da freqüência de portadores e da aceitação desse programa pela comunidade.

**LOCAL:**

Laboratório de genética molecular, Instituto de Biociências, Universidade de São Paulo.

**PARTICIPANTES:**

581 alunos do 3°. ano do ensino médio de escolas judaicas selecionadas.

**PROCEDIMENTOS:**

Análise molecular de mutações causadoras da doença de Tay-Sachs através de amplificação de DNA genômico por PCR e digestão por enzimas de restrição.

**RESULTADOS:**

Entre 581 alunos que assistiram palestras informativas, 404 (70%) decidiram realizar o teste para detecção de portadores. Desses, aproximadamente 65% tinham origem judaica Ashkenazita. Oito portadores foram identificados correspondendo a uma freqüência de portadores de um em cada 33 indivíduos na fração judaica Ashkenazita da amostra.

**CONCLUSÕES:**

A freqüência de portadores da doença de Tay-Sachs, na população judaica Ashkenazita brasileira, é similar àquela de outros países nos quais programas de triagem levaram a uma redução significativa na incidência da doença. Assim, é justificável a implementação de um programa de triagem de portadores da doença de Tay-Sachs na população judaica brasileira.

## INTRODUCTION

Tay-Sachs disease is an autosomal recessive disease of lysosome storage characterized by progressive neurologic degeneration.^1^ Children affected by classic Tay-Sachs disease manifest the first symptoms at around 6 months and die before reaching 5 years of age. The clinical manifestations are particularly severe including deafness, blindness, dementia, and recurrent convulsions during the terminal stage when affected children are confined to bed. There is currently no treatment available.

Tay-Sachs disease is caused by mutations in the HEXA gene, located at 15q23-q24, which codes for the alpha subunit of the hexosaminidase A enzyme.^2^^,^^3^ In the absence of the enzyme, its substrate, G_M2_ ganglioside, accumulates progressively in the neurons of the central nervous cortex leading to the clinical phenotype of the disease. Late-onset Tay-Sachs disease (chronic form) is a rare variant phenotype with appearance of first symptoms during the second or third decade of life.^4^ A juvenile form is also distinguished, with an intermediate presentation. The less severe phenotypes are due to residual enzyme activity.^5^

As observed for several recessive traits, Tay-Sachs disease incidence concentrates in some specific populations. That is the case for the Ashkenazi Jewish population (Jews of Central or Eastern Europe descent), in which the disease incidence is 1 in every 3,500 newborns: approximately 100 times higher than in the general population. Among Ashkenazi Jews, 1 in every 29 individuals is heterozygous (asymptomatic) for Tay-Sachs disease causing mutations (here called carriers).^6^

Following the development of prenatal diagnosis for Tay-Sachs disease in the early 1970's,^7^ most couples who had had an affected child chose to monitor subsequent pregnancies and bring to term only pregnancies of unaffected fetuses. Other options for carrier couples include adoption, sperm or egg donation, pre-implantation diagnosis, reproductive abstention or simply taking their 25% risk.

Since only couples with a previous affected child could be aware of their risk, carrier screening programs were massively initiated in the high-risk populations, aiming at detecting and informing carrier couples prior to any family history of the disease. These programs were made possible due to the development of an enzymatic assay that allowed the detection of heterozygotes for Tay-Sachs disease.^8^^,^^9^

Up to 1992, over one million individuals had been tested for Tay-Sachs disease carrier status, more than 36,000 carriers had been detected and 1,054 carrier couples had been identified and informed of their risk prior to having an affected child. These programs led to a 90% decrease in the incidence of Tay-Sachs disease in the Ashkenazi Jewish populations in the USA, Israel and Canada.^10^

Molecular diagnosis for Tay-Sachs disease in specific populations has presented a complementary or even alternative way for performing enzymatic assay in detecting carriers. Three mutations are responsible for 98% of the disease incidence in the Ashkenazi Jewish population.^11^^,^^12^ A 4-base pair insertion in exon 11 of the HEXA gene (InsTATC1278) is present in 80% of Tay-Sachs disease, causing alleles.^13^ The second most frequent mutation leading to Tay-Sachs disease in Ashkenazi Jews is a guanine to cytosine transvertion in the first base of intron 12 (IVS12+1), and is present in 16% of the alleles.^14^^,^^15^ The third and most rare mutation, present in 2% of Tay-Sachs disease cases, is a guanine to adenine transition in the last nucleotide of exon 7 leading to a substitution of glycine for serine in position 269 of the alpha subunit of the hexosaminidase A enzyme (Gly269Ser). This last mutation leads to late-onset Tay-Sachs disease in the Ashkenazi Jewish population.^4^^,^^16^

Brazil has the 8^th^ largest Jewish population in the world, counted as approximately 90,000 individuals in 1991.^17^ This work was designed to evaluate the need for and acceptance of a screening program for Tay-Sachs disease carriers in the Brazilian Jewish population. For that purpose, we evaluated the frequency of carriers in a sample of this population and observed the reaction of the community to the proposal of a pilot screening program for Tay-Sachs disease carriers.

## METHODS

During the last three years, educational classes on Tay-Sachs disease were presented to senior students at five Jewish high schools, three in the city of São Paulo (1998, 1999 and 2000) and two in the city of Rio de Janeiro (1999 and 2000). These institutions are the main non-orthodox Jewish high schools of these cities. The students were 16 years of age and over, except for some participants from Colégio Liessin (2000), where the pre-senior class attended the program at the school's request.

After the presentation, students took home a consent form with a brief explanation of the program, the possible results of the test and a questionnaire for the parents. The questionnaire was aimed at evaluating the impact of similar programs from other countries (whether they would serve as an indication for Tay-Sachs disease testing among Brazilians), the ethnic origin of the students (Ashkenazi or not) and the acceptance of the program.

About a week later and with parents’ consent, students who elected to be tested had mouth mucus collected by twirling a cotton swab on the inner cheek for a few seconds. The material was then taken to the laboratory where DNA was extracted according to Richards et al., 1993.^18^

Detection of mutations InsTATC1278, IVS12+1 and Gly269Ser was performed by PCR amplification of genomic DNA and subsequent digestion with restriction enzymes as described elsewhere.^11^

The results and their interpretation were confidentially sent by mail to the students. All samples and results were identified with codes so that a direct connection between student name and test result could not be made.

The carrier frequencies obtained were compared to those from other studies using Fisher's exact test. The statistical difference between two samples was considered non-significant when P > 0.05.

## RESULTS

From 1998 to 2000, thirteen educational sessions were attended by 581 students. Of these students, 404 came to the screening sessions and delivered the consent form, an overall participation rate of 70%.

Table 1 summarizes the participation rates by year in each school. Chi-squared analysis showed that the participation rates did not differ significantly among the schools (P = 0.52), over the years (P = 0.22) and between the cities of São Paulo and Rio de Janeiro (P = 0.71).

**Table 1:** Participation rates* of senior students from selected Jewish high schools

Year	School name (City) **	Students		Participation Rate*
		Attended	Participated	
1998	Bialik (SP)	41	21	51%
	Peretz (SP)	31	20	65%
	Renascença (SP)	39	26	67%
*sub-total*		111	67	60%
1999	Bialik (SP)	48	36	75%
	Peretz (SP)	43	37	86%
	Renascença (SP)	27	23	85%
	Eliezer (RJ)	35	24	69%
	Liessin (RJ)	36	26	72%
*sub-total*		189	146	77%
2000	Bialik (SP)	43	25	58%
	Peretz (SP)	37	30	81%
	Renascença (SP)	57	40	70%
	Eliezer (RJ)	49	38	78%
	Liessin (RJ)	95	58	61%
*sub-total*		281	191	68%
**TOTAL**		**581**	**404**	**70%**

* Participation was defined as the student's presence at the screening session and delivery of the completed consent form.

** SP: São Paulo, RJ: Rio de Janeiro.

In order to access parents’ opinions on the importance of the program, they were asked in the consent form: "How would you classify this program?". The results are shown in Table 2. The program was rated as "important", "very important" or "essential" in 95% of the cases, among the parents of participating students.

**Table 2:** Participants’ parents’ opinions on the program

Opinion	n	(%)
Negative	0	0%
Insignificant	1	0%
Not very important	5	1%
Important	184	28%
Very important	274	42%
Essential	165	25%
Not responded	20	3%
**Total**	**649**	**100%**

3 n: number of parent replies, among consent forms for the 404 participants; (%): percentage of the total number of answers (including non-respondents).

In order to estimate the Ashkenazi fraction of the sample, we asked in the consent form about the possibility of Ashkenazi ancestry and the country of origin of the student's grandparents (data not shown). From these data it was readily apparent that 26% of the students had no Ashkenazi Jewish ancestry. For the remaining students, the grandparents’ country of origin allowed us to estimate that 65% of the students’ chromosomes were associated with Ashkenazi origin. This estimate is close to others previously established for the Brazilian Jewish population.^19^^,^^20^

Among the 404 participants, eight carriers were found. All the carriers had Ashkenazi Jewish ancestry. Of the eight mutations detected, seven were InsTATC1278 and one was IVS12+1. This indicates a carrier frequency of 1 in every 51 students, similar to that observed in several Jewish populations (P = 0.16).^10^

When the carrier frequency was separated by state, we found 7 carriers among 258 participants in São Paulo and 1 carrier among 146 participants in Rio de Janeiro. The difference in the carrier frequency between these two cities was not significant (P= 0.27).

When corrected for the Ashkenazi fraction of the sample, which comprised 65% of the 404 students’ chromosomes, the estimated carrier frequency for Tay-Sachs disease is 1 in 33 (8/263), similar to that observed in other Ashkenazi Jewish populations (P = 0.87).^6^

During the study, three carriers contacted the lab for re-testing and additional counseling. The mother of a carrier was also identified as a carrier.

## DISCUSSION

One of the necessary prerequisites for the establishment of a carrier screening program for a recessive genetic disease is a high carrier frequency in the target population. It is also important to confirm the carrier frequency in similar populations from different countries since different migration patterns or different admixture rates could lead to populations with similar origin, but different carrier frequencies. For instance, the incidence of Tay-Sachs disease among individuals of French-Canadian heritage living in northern New England (USA) is lower than that observed among those living in Quebec (Canada), downplaying the need for a screening program in the former.^21^

In contrast, the carrier frequency of Tay-Sachs disease mutations in the Brazilian Jewish individuals herein studied is similar to that observed in the Jewish population of several other countries. The high carrier frequency found in the Brazilian Jewish population indicates the need of a screening program for Tay-Sachs disease in Brazil like those effectively developed in other Jewish communities for over 30 years.

The 70% participation rate shows good acceptance of the screening program by the community. This was also demonstrated by the participant's parents’ rating of the program as "important", "very important" or "essential" in 95% of the cases, and the absence of a significant decrease in the participation rates in the years following the first presentations (Table 1). Among the factors that may have led to the high participation rate in our study were the participants’ easy access to screening locations; a collection method that did not involve blood extraction; and finally, a higher knowledge of genetics in general due to the exposure in the media that the Human Genome Project has had in recent years.

The Tay-Sachs disease screening program was the first to be performed for a genetic disease and served as a prototype for other preventive programs.^23^ Currently, over 50 centers in the world promote Tay-Sachs disease carrier screening. Although notable community mobilization has been obtained by some programs, others have not readily reached the same success. Such was the case in Canada, where researchers decided to move into the community institutions in order to reach participants instead of waiting for self-mobilization to screening centers.

The Canadian Tay-Sachs disease program has been operating for over 20 years, screening senior high schools students and obtaining a similar participation rate (67%) to the one in this study (70%).^23^ Initially, its approach to screening faced the ethical controversy of testing single teenagers.^24^ A follow-up study of the program showed that most students had positive attitudes after screening and that both carriers and non-carriers considered the test during high school as not being premature.^25^ The Canadian program served as a model for the research presented here, since two previous attempts to establish preventive programs in Brazil were discontinued.^26^^,^^27^ Before we directed it towards high schools, the offer of a screening program was made to different Jewish institutions. Although approval was obtained in some instances, the only institutions that presented a plausible approach to screening were the non-orthodox Jewish high schools.

Besides the targeting of the screening, other ethical aspects were an essential part of this pilot program. In particular, we avoided stigmatization of carriers by focusing on delivering precise and comprehensive information about Tay-Sachs disease to students. Additionally, in order to maintain maximum confidentiality of test results, especially important in small communities, samples and results were coded, and the results were delivered to the participants by mail. This procedure prevented the personal identification of carriers, even by researchers, unless they contacted the laboratory. Most students (91%) affirmed that they would prefer to get a positive result by mail rather than by a phone call.

Data from the consent form also indicated that the success of screening programs in other countries does not diminish the need for a program in Brazil. Only 15% of students’ parents affirmed they had relatives living in USA, Israel or Canada, the principal countries in which preventive programs are conducted. Only 2% (11/651) of the parents had already been tested for Tay-Sachs disease, none of whom because of recommendation by foreign relatives.

Finally, this was a pioneering piece of research in that it exclusively used molecular diagnosis to detect Tay-Sachs disease carriers. It has corroborated the prediction that "the development of molecular diagnosis allows the performance of the DNA test in a small center without the need to maintain the rigorous quality control required for the enzyme as- say".^11^ The molecular diagnosis also permits the tests to be performed on mouth mucus collection instead of requiring blood testing. However, while DNA test is adequate for Ashkenazi Jews, the enzyme assay is more appropriate for the general population since different mutations may be present.^28^

In addition to the continuity of this screening program for Tay-Sachs disease carriers, an ideal outcome from this research would be to bring it to the attention of physicians assisting Brazilian Jewish couples that they may be attending a Tay-Sachs disease carrier couple. It is important to note that the carrier frequency of Tay-Sachs disease among the general population is about 10 times lower than in the population at risk, significantly diminishing but not eliminating Tay-Sachs disease among non-Jews. Recently, due to intermarriage and declining awareness of ancestry, screening has been recommended even for couples for whom only one member is thought to have ancestry in a high-risk population.^28^ Screening can confirm or exclude the possibility of both members of a couple being Tay-Sachs disease carriers, and is the best indication for prevention until an effective treatment becomes available for this disease.

## CONCLUSION

The carrier frequency of the main mutations causing Tay-Sachs disease in senior students of Jewish high schools was 1/51. The corrected carrier frequency of Tay-Sachs disease in the Ashkenazi Jewish fraction of the sample was 1/33, similar to that in Ashkenazi Jewish populations of other countries. The voluntary participation rate in a pilot screening program of Tay-Sachs disease in Jewish high schools between the years 1998 and 2000 was 70%.

Based on the efficiency of screening programs for Tay-Sachs disease in alerting carrier couples and decreasing the disease incidence in other countries, a similar program should be implemented for the Brazilian Jewish population. In addition, physicians should recommend testing for Tay-Sachs disease mutations for individuals of Ashkenazi Jewish ancestry at reproductive age.
